# Semi-Automatic Calibration Method for a Bed-Monitoring System Using Infrared Image Depth Sensors

**DOI:** 10.3390/s19204581

**Published:** 2019-10-21

**Authors:** Hideki Komagata, Erika Kakinuma, Masahiro Ishikawa, Kazuma Shinoda, Naoki Kobayashi

**Affiliations:** 1 Faculty of Health and Medical Care, Saitama Medical University, 1397-1 Yamane, Hidaka, Saitama 350-1241, Japan; 2 Shin-Oyama City Hospital, 2251-1 Hitotonoya, Oyama, Tochigi 323-0827, Japan; 3 School of Engineering, Utsunomiya University, 7-1-2 Yoto, Utsunomiya, Tochigi 321-8585, Japan

**Keywords:** patient monitoring, bed-monitoring system, camera calibration, 3D point cloud, plane detection, depth sensors, infrared-image sensors

## Abstract

With the aging of society, the number of fall accidents has increased in hospitals and care facilities, and some accidents have happened around beds. To help prevent accidents, mats and clip sensors have been used in these facilities but they can be invasive, and their purpose may be misinterpreted. In recent years, research has been conducted using an infrared-image depth sensor as a bed-monitoring system for detecting a patient getting up, exiting the bed, and/or falling; however, some manual calibration was required initially to set up the sensor in each instance. We propose a bed-monitoring system that retains the infrared-image depth sensors but uses semi-automatic rather than manual calibration in each situation where it is applied. Our automated methods robustly calculate the bed region, surrounding floor, sensor location, and attitude, and can recognize the spatial position of the patient even when the sensor is attached but unconstrained. Also, we propose a means to reconfigure the spatial position considering occlusion by parts of the bed and also accounting for the gravity center of the patient’s body. Experimental results of multi-view calibration and motion simulation showed that our methods were effective for recognition of the spatial position of the patient.

## 1. Introduction

An aging population makes up a progressively larger proportion of society in developed countries in recent years. With this trend, the number of accidents involving falls has increased in hospitals and other care facilities [1,2,3,4]. Because possibly delayed discovery of such accidents is a life-threatening risk, their early detection is important for these facilities. Some fall accidents happen during unmonitored walking. For early detection of these accidents, fall-detection systems for use during walking have been studied over the last few years [5,6,7,8,9,10,11,12]. The researchers used various devices, such as RGB cameras, depth sensors, infrared sensors, and accelerometers.

Some fall accidents also have happened around beds, and fall-risk assessment tools have been used to evaluate the risk of accidents involving falling from bed [13,14]. If a patient has been evaluated as having a high risk of fall accidents, risk-mitigating measures are taken; these include attaching a sensor to the patient to detect when they get up or when they get out of bed [15]. The main sensors in current use to monitor patients are clip sensors that attaches to clothing, floor-mat sensors, and bed-mat sensors [16,17]. The clip sensor sounds an alarm when the clip is pulled. It is inexpensive but invasive. The floor- and bed-mat sensors are noninvasive but occasionally do trigger false alarms, which are burdens for medical staff [15]. Various sensors have been tested in recent years in an attempt to reduce the false alarms. In some studies, multiple piezoelectric elements [18,19] or strain gauges [20] placed on the bed recognized the position of the patient in the bed, while some other studies used ultrasonic or infrared depth sensors [21,22,23,24] to recognize the position of the patient both in bed and out of bed. In our study, we used an infrared depth sensor, which can obtain a wide range of depth information as a depth image and can monitor patients day or night.

Some conventional methods using infrared depth sensors installed vertically from the ceiling to the floor detect bed-exit and fall events by determining the position of the patient from the depth image [22,25]. Although this simplifies the calculation of the position, installation work is required. Although other conventional methods do not require ceiling installation of the sensors [23,24,26], they have other drawbacks. The method proposed by Asano et al. [23] is limited to use under the condition that only the bed and wall (not the floor) are imaged by the sensor. Methods by Ogura et al. [24] and by Ni et al. [26] both require manual intervention to identify the part of the captured depth image that includes the bed, and this laborious initial calibration is required every time the position of the sensor or bed is changed.

Beyond simply detecting the bed region, other researchers have studied several alternative methods that recognize spaces from a three-dimensional (3D) point cloud. Banerjee et al. [12] devised a method that automatically detects the floor surface and recognizes floor-level falls by applying the dense scale-invariant feature-transform (dense SIFT) method [27] and the random-sampling consensus (RANSAC) algorithm [28] to the depth image. Nurunnabi et al., Limberger et al., and Vera et al. studied methods for detecting planes from 3D point-cloud data [29,30,31]. In particular, Vera et al. [31] proposed a specialized plane-detection method that combines range images with principal component analysis (PCA) and 3D Hough transform. Although this method, known as the depth kernel-based Hough transform (D-KHT) method, is fast and robust, because it was not designed for bed monitoring, it does not calculate either the bed region or the sensor position and attitude (an angle of rotation).

We propose a bed-monitoring system that incorporates a semi-automatic initial-calibration method using an infrared depth sensor attached in any position without installation on the ceiling. This method automatically calculates the bed region, sensor position and attitude, and a spatial domain for recognizing various behaviors such as sitting up in bed or getting out of bed. The floor and bed surfaces are planar features in the space analyzed, therefore we extract them using PCA and k-means++ clustering [32]. There is also a difference in level between the floor and bed surfaces in the real space, and the boundary appears as an edge on the depth image. We therefore distinguish between the floor and bed regions using Canny’s edge-detection method [33,34]. Then, geometric calculation based on principal component axes of the bed region automatically yield the sensor position and attitude and the spatial position of the patient (see Section 2.2; we previously reported this approach in part at a forum in 2017 [35]). Alternatively, the floor and bed regions can be extracted by the D-KHT method (see Section 2.3). We compare the precision and calculation speeds of the D-KHT based depth sensor calibration (DDC) and PCA based depth sensor calibration (PDC) methods in Section 3.

We also took into consideration error factors such as occlusion and gravity-center misalignment. When the bed is not directly under the sensor, part of the bed region on the image is hidden by the head of the bed, resulting in occlusion. Therefore, when the depth sensor measures the 3D position of the surface of an object such as the patient, the person’s position as acquired by the sensor may be slightly different from the actual gravity center of the patient. Because these occlusion and misalignment error factors affect actual monitoring, we propose the following correction methods:

- Installation of the depth sensor in any position and attitude without installation on the ceiling, such as on a tripod or on a bed fence. However, both the bed and floor need to be captured and their total area needs to be larger than the wall area.
- Automatic calculation of the bed and floor regions, sensor position and attitude, and spatial domain.
- Reconfiguration of the spatial domain considering occlusion by the head or foot of the bed.
- Reconfiguration of the spatial domain considering the gravity center of the patient.

Among these four, the automatic calculations of regions are incorporated into the PDC and DDC methods. Our system automatically calculates a series of processes for the above initial calibration. However, calibration may fail depending on the installation conditions; therefore, it is necessary to visually check the calibration results and, in some cases, re-install.

## 2. Calibration Methods

### 2.1. System Configuration

**Figure 1:**
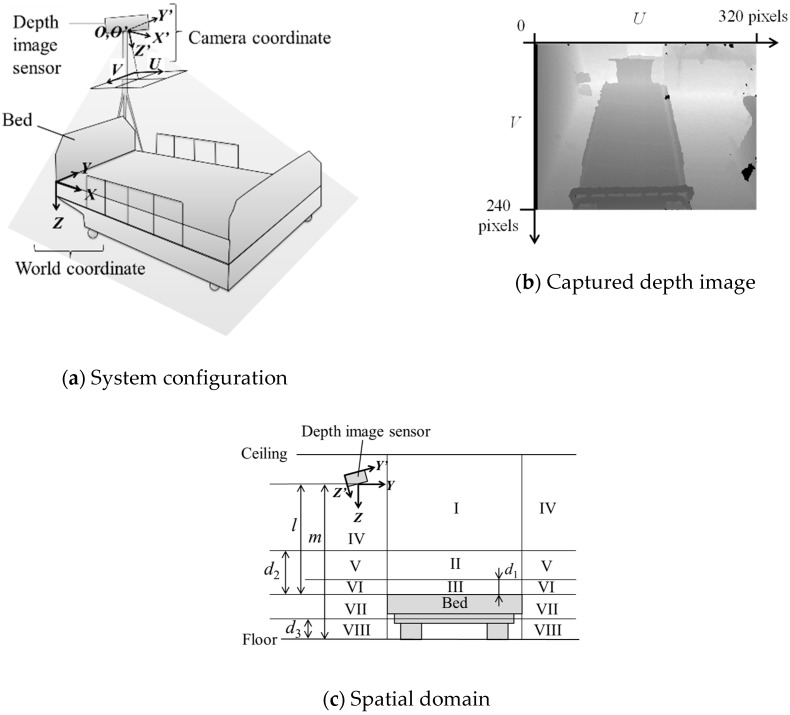
System configuration and captured depth image.

The initial-calibration method of the bed-monitoring system uses an infrared depth sensor installed in any position and attitude. As shown in Figure 1a, ***X***, ***Y***, and ***Z*** denote the length, width, and height axes of the bed surface in the world coordinate system, respectively, and the ***X’***, ***Y’***, and ***Z’*** axes denote the horizontal, vertical, and optical axes in the sensor coordinate system, respectively. Both the origin ***O*** and ***O’***, which respectively indicate the world and sensor coordinate systems, were set as the focal position of the sensor.

The infrared depth sensor includes an infrared projector and an infrared camera. It can measure the distance from the sensor to the object, i.e., the *Z’* value of the object, by time of flight (TOF) or triangulation. Figure 1b is a depth image drawn by converting linearly so as to become brighter as *Z’* value increases using an infrared depth sensor XTION PRO of ASUSTeK Computer Inc. Black regions with intensities 0 of Figure 1b are defective regions where we could not acquire depth data. The resolution of the XTION PRO is 320 × 240 pixels, and 3D point cloud data (76,800 points) can be acquired simultaneously.

Figure 1c is a schematic of the spatial domain; axes components ***Y*** and ***Z*** correspond to those in Figure 1a. The space is divided into eight regions (I to VIII) with reference to the bed boundary and preset threshold values *d*_1_ to *d*_3_. Threshold *d*_1_ is a height from the bed chosen to discern whether or not the patient is in the bed, and threshold *d*_2_ is a height from the bed to discern whether the patient is in a sitting or a sleeping position. The *d*_3_ is a height threshold from the floor to distinguish whether or not the patient (or an object) is on the floor. If nobody is on the bed, the collected 3D points data do not include region I or II. However, if the patient is asleep on the bed, the 3D points include region II, and if the patient is sitting up on the bed, the 3D points also include region I. In addition, if the patient leaves the bed, the 3D points include regions IV to VII, and if the patient falls on the floor, the 3D points also include region VII and VIII. Previously, some conventional methods detected a patient’s getting up by monitoring regions I and II and detected a patient’s getting out of bed or falling by monitoring regions IV to VII [22,23].

In order to construct such a bed-monitoring system as we propose, it is necessary to recognize the bed region on the captured image. If the sensor is installed in any position, it is also necessary to recognize the position and attitude of the sensor. In practice, a simple method with an easy initial setup is desirable. In the next two sections, we describe two initial-calibration methods, PDC and DDC, for automatically calculating the bed region and the sensor position and attitude. We also describe how to reconfigure the spatial domain while considering occlusion by the head of the bed and the gravity center of the patient in the later Section 2.4, and finally, we describe the spatial-domain monitoring method using these in Section 2.5.

### 2.2. PCA-Based Depth Sensor-Calibration (PDC) Method

The relationship between the captured-image coordinate (u,v) and sensor coordinates ${P^{\prime }}_{u,v}$ is expressed as

(1) $${P^{\prime }}_{u,v}=sI_{u,v}\left[\begin{array}{c} a\left(u-C_{u}\right)\\ -a\left(v-C_{v}\right)\\ 1 \end{array}\right],$$

where *I_u_*_,*v*_ is an intensity of coordinate (u,v); *s* is a depth interval per unit intensity on ***Z’*** axis, *C_u_* and *C*_v_ are the ***U*** and ***V*** axis coordinates of the optical center, in pixel; and *a* is a ratio between the focal length and physical pixel size of the sensor. According to the sensor specifications, we used *s* = 1.6 cm/intensity, (*C_u_*, *C_v_*) = (160, 120), and *a* = 0.003452.

Normally, the bed and floor surfaces are flat in real space, and both are the same gradient. We therefore calculate the object gradient in the sensor coordinate system as ${N^{\prime }}_{u,v}$, the unit normal vector of the surface of the object whose coordinate on the image are (u,v), by

(2) $${N^{\prime }}_{u,v}=f_{\mathrm{PCA}3}\left\{{P^{\prime }}_{i,j}|u-b\leq i\leq u+b\cap v-b\leq j\leq v+b\cap I_{u,v}>0\right\},$$

where *f*_PCA3_ is a function to calculate the unit eigenvector of the third principal component (the third largest eigenvalue of the covariance matrix); and *b* is the range of neighboring pixels used for the gradient calculation (we used the empirical value *b* = 5 pixels). Because ${N^{\prime }}_{u,v}$ appears as two vectors with the point symmetry, we use a vector closer to ${P^{\prime }}_{u,v}$ such that $\left({N^{\prime }}_{u,v}\cdot {P^{\prime }}_{u,v}\right)>0$. The condition $I_{u,v}>0$ means that defective pixels are excluded.

**Figure 2:**
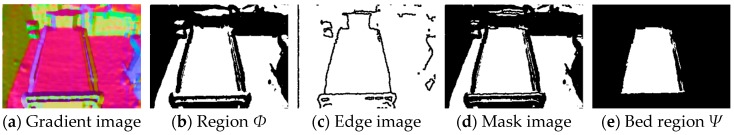
Extraction of bed region using the principal component analysis-based depth-sensor calibration (PDC) method.

${N^{\prime }}_{u,v}$ represents the gradient of the coordinates (u,v) in 3D space. We calculate ${N^{\prime }}_{u,v}$ for the all pixels on captured image using Equation (2) and make a gradient image using by

(3) $$\left[\begin{array}{c} J_{R_{u,v}}\\ J_{G_{u,v}}\\ J_{B_{u,v}} \end{array}\right]=\frac{254}{2}\left({N^{\prime }}_{u,v}+1\right),$$

where $J_{R_{u,v}}$, $J_{G_{u,v}}$, and $J_{B_{u,v}}$ are the red, green, and blue intensities in the coordinate (u,v), respectively, and each of these three intensities is in the range of 0 to 254. Figure 2a shows the gradient image of the captured depth image (Figure 1b) visualized by Equation (3). Equation (3) is an equation for linearly rescaling ${N^{\prime }}_{u,v}$ in which each element is in the range of −1 to 1 to a color image in which each element is in the range of 0 to 254. It is used only for visualization in this paper and debugging of this system, not directly for calibration.

As shown in Figure 2a, the bed and floor regions are the same color, indicating that these surfaces are the same gradient in real 3D space. In addition, these regions occupy a large area on the image. We therefore using k-means++ algorithm [32] to clustered ${N^{\prime }}_{u,v}$; we set the number of clusters *k* = 4 and extracted the class with the largest area. The horizontal plane region *Φ* is defined by pixels belonging to this largest class. The result of extracting *Φ* from the captured depth image (Figure 1b) is shown in Figure 2b.

The region *Φ* includes the bed, floor, and other horizontal surfaces such as the upper surface of the shelf. These boundaries usually have steps, and in the case of depth images, the steps appear as edges with high-intensity gradients. To distinguish these regions, we extract the edges by applying the widely used Canny algorithm [33,34] and taking a mask (logically multiplying) of *Φ* and the edges. Figure 2c,d shows the results of detecting the edge of the captured depth image (Figure 1b) and the mask image, respectively. The Canny algorithm requires edge extraction thresholds *T*_1_ and *T*_2_ and a parameter σ. These will be examined in the pre-experiment in Chapter 3. Note that *T*_1_, *T*_2_, and σ in Figure 2c were calculated as 50, 100, and 3, respectively. Although the bed and floor surfaces are partially connected in the lower part of Figure 2b, they are separated in Figure 2d.

Next, we extract only the bed region from the mask image. In this method, since the bed surface is the monitoring target, that surface area is normally the largest in the mask image. We therefore extract only the bed region by labeling the mask image and extracting the label with the maximum area. Figure 2e shows the result of extracting the bed region, *Ψ*, from the captured depth image (Figure 1b).

Then, using the extracted horizontal-plane region *Φ* and the bed region *Ψ*, we automatically calculate the distance *l* between the sensor and the bed surface and the distance *m* between the sensor and the floor (Figure 1c). We also automatically calculate the sensor attitude (an angle of rotation of the sensor) ***R*** which is 3 × 3 matrix that relates the world coordinate system ***XYZ*** to the sensor coordinate system ***X’Y’Z’*** as

(4) $${\left[\begin{array}{ccc} X & Y & Z \end{array}\right]}^{⊤}=R{\left[\begin{array}{ccc} X^{\prime } & Y^{\prime } & Z^{\prime } \end{array}\right]}^{⊤}.$$

When PCA is performed again on the sensor coordinate values ${P^{\prime }}_{u,v}$ of the pixels in the bed region *Ψ*, the eigenvectors of the first, second and third principal components respectively indicate the bed length (long axis), bed width (short axis), and the gradient of the bed surface in the sensor coordinate system. Because these directions are defined as ***X***, ***Y***, and ***Z*** axes in the world coordinate system, ***R*** can be obtained by

(5) $$R=f_{PCA}\left\{{P^{\prime }}_{u,v}|\left(u,v\right)\in \Psi \right\},$$

where *f_PCA_* is a function to calculate the unit eigenvectors of the three principal components.

From the calculated sensor attitude ***R***, world coordinate value ***P****_u_*_,*v*_ of the image coordinates (u,v) can be derived by

(6) $$P_{u,v}=\left[\begin{array}{c} X_{u,v}\\ Y_{u,v}\\ Z_{u,v} \end{array}\right]=R{P^{\prime }}_{u,v},$$

where *X_u_*_,*v*_, *Y_u_*_,*v*_, and *Z_u_*_,*v*_ are respectively defined as *X*, *Y*, and *Z* components of ***P****_u_*_,*v*_. Given that each eigenvector in Equation (5) appears as two vectors with the point symmetry, we used the vector where *Z_u_*_,*v*_ > 0.

In the bed region *Ψ*,

(7) $$\left[\begin{array}{c} \tilde{X}\\ \tilde{Y}\\ \tilde{Z} \end{array}\right]=\left\{P_{u,v}|\left(u,v\right)\in \Psi \right\},$$

where $\tilde{X}$, $\tilde{Y}$, and $\tilde{Z}$ correspond to the set of *X_u_*_,*v*_, *Y_u_*_,*v*_, and *Z_u_*_,*v*_ (lengths, widths, heights in the world coordinate system of 3D points on the bed surface). We calculate a height from the bed surface to the sensor (Figure 1c), *l*, by

(8) $$l=f_{Ave}\left(\tilde{Z}\right),$$

where *f_Ave_* is a function to obtain the average value.

In addition, many pixels in the horizontal surface region *Φ* excluding the bed surface region *Ψ* represent the floor surface. However, since it does not represent only the floor surface, *m* is calculated by

(9) $$m=f_{Mode}\left(Z_{u,v}|\left(u,v\right)\in \Phi \cap \left(u,v\right)\notin \Psi \right),$$

where $f_{Mode}$ is a function to obtain the mode value. The mode is calculated by making a histogram with interval width *τ* and extracting the value with the maximum frequency. We set *τ* = 0.1 cm in consideration of the resolution of the sensor. Details of the spatial domain division (Figure 1c) using these parameters are described in Section 2.5.

### 2.3. D-KHT Based Depth-Sensor Calibration (DDC) Method

**Figure 3:**
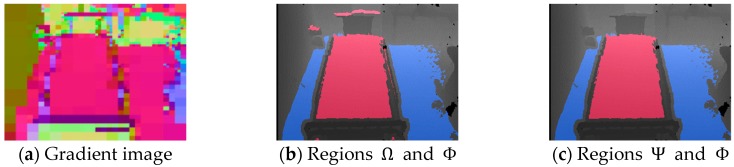
Extraction of bed region by depth sensor initial-calibration by high density (DDC-D) method.

We based our depth sensor initial-calibration method (DDC) on the D-KHT method proposed by Vera et al. [31]. They also calculated the gradient of the depth image using Equation (1) and PCA. However, they computed recursively and in block units using Quadtree instead of incorporating all pixel calculations as we proposed in Section 2.2. Their method, using Quadtree, can calculate faster than the method that uses all pixels. They used Quadtree’s recursive division criterion (*s_t_*) as the square root of the third eigenvalue of PCA (i.e., the standard deviation of the distance between the plane and the point). Figure 3a shows the result of calculating the gradient of ***Q****_i_* as ***N****_i_* = (***N****_i_*_,*x*_, ***N****_i_*_,*y*_, ***N****_i_*_,*z*_) of the image (Figure 1b), where the *i*^th^ Quadtree is ***Q****_i_*, *s_t_* = 2 cm, and color coding is based on Equation (3).

Vera et al. [31] then converted ***N****_i_* to 3D polar coordinates ***μ****_i_* = (*μ_i_*_,*ρ*_, *μ_i,φ_*, *μ_i_*_,*θ*_) using Equation (10) and voted the 3D voxel space *W_ρ_*_,*φ*,*θ*_ in consideration of the area of Quadtree and the probability density function (PDF) of the Gaussian distribution.

(10) $$\mu_{i}=\left[\begin{array}{c} \mu_{i,\rho }\\ \mu_{i,\varphi }\\ \mu_{i,\theta } \end{array}\right]=\left[\begin{array}{c} N_{i}\cdot M_{i}\\ \cos^{-1}\left(N_{i,z}\right)\\ \tan^{-1}\left(\frac{N_{i,y}}{N_{i,x}}\right) \end{array}\right],$$

where $M_{i}$ is the average value of ${P^{\prime }}_{u,v}$ in the Quadtree; and *S_ρ_*, *S_φ_*, and *S_θ_* are voxel sizes of *ρ*, *φ*, and *θ* in the *W_ρ_*_,*φ*,*θ*_, respectively. Subsequently, they smoothed the *W_ρ_*_,*φ*,*θ*_ with a low-pass filter and used the polar coordinates (*ρ*, *φ*, *θ*) of the local maximum of the *W_ρ_*_,*φ*,*θ*_ as planes.

Because Vera et al. [31] aimed at plane extraction, not bed-region extraction, we propose a method to extract bed- and floor-region candidates by the following procedure of Section 2.3.1, Section 2.3.2, Section 2.3.3, Section 2.3.4, Section 2.3.5 and Section 2.3.6.

#### 2.3.1. Step 1: Calculation of Horizontal-Plane Polar Coordinates $\varphi^{\prime }$ and $\theta^{\prime }$

The bed and floor surfaces are the same gradient. That means the polar coordinates (φ,θ) of the bed surface are equal to the polar coordinates (φ,θ) of the floor surface. They also occupy a large area in the image. Therefore, we respectively set the mode values of φ and θ as the horizontal-plane polar coordinates $\varphi^{\prime }$ and $\theta^{\prime }$ which represent the bed or floor surfaces. Using the same voxel voting space $W_{\rho ,\varphi ,\theta }$ as Vera et al. [31], these are calculated by

(11) $$\left(\varphi^{\prime },\theta^{\prime }\right)=\underset{\left(\varphi ,\theta \right)}{\mathrm{argmax}}\left(\sum_{\rho }W_{\rho ,\varphi ,\theta }\right).$$

#### 2.3.2. Step 2: Calculation of the Distance between the Sensor and Floor $\rho^{\prime }$

We set the voxel space of coordinate $\left(\varphi^{\prime },\theta^{\prime }\right)$ as a horizontal-plane space $W_{\rho ,\varphi^{\prime },\theta^{\prime }}$ and set the distance between the sensor and the floor calculated in this procedure as $\rho^{\prime }$. We set the local maximum value of $W_{\rho ,\varphi^{\prime },\theta^{\prime }}$ farthest from the sensor as $\rho^{\prime }$.

#### 2.3.3. Step 3: Calculation of Distance $\rho^{\prime \prime }$ between the Sensor and the Bed

We set the distance between the sensor and bed calculated in this procedure as $\rho^{\prime \prime }$. Assuming that the bed surface is a large area between the floor surface and the sensor, $\rho^{\prime \prime }$ is calculated by

(12) $$\rho^{\prime \prime }=\underset{\rho }{\mathrm{argmax}}\left(W_{\rho ,\varphi^{\prime },\theta^{\prime }}|0<\rho <\rho^{\prime }-\delta \right),$$

where δ, given as a constant in advance, is the lower limit of the height of the bed (to prevent erroneous extraction). The polar coordinates $\left(\rho^{\prime \prime },\varphi^{\prime },\theta^{\prime }\right)$ represent the bed surface.

#### 2.3.4. Step 4: Calculation of the Floor Surface Φ and Bed-Region Candidate Ω

In this procedural step, we propose two alternatives for calculating the floor surface Φ and bed-region candidate Ω. The first (DDC-D) calculates the bed region with high-density pixel units, and the second (DDC-S) calculates the bed region at high speed in block units of the Quadtree. For both, extraction thresholds $\eta_{\rho }$, $\eta_{\varphi }$, and $\eta_{\theta }$ are given as constants in advance.

##### Alternative 1: DDC Method with High Density (DDC-D)

For all pixels in the image, gradients ${N^{\prime }}_{u,v}=\left({N^{\prime }}_{u,v,x},{N^{\prime }}_{u,v,y},{N^{\prime }}_{u,v,z}\right)$ are calculated by using Equation (2) and polar coordinates $\nu_{u,v}$ are calculated by

(13) $$\nu_{u,v}=\left[\begin{array}{c} \nu_{u,v,\rho }\\ \nu_{u,v,\varphi }\\ \nu_{u,v,\theta } \end{array}\right]=\left[\begin{array}{c} {N^{\prime }}_{u,v}\cdot {P^{\prime }}_{u,v}\\ \cos^{-1}\left({N^{\prime }}_{u,v,z}\right)\\ \tan^{-1}\left(\frac{{N^{\prime }}_{u,v,y}}{{N^{\prime }}_{u,v,x}}\right) \end{array}\right].$$

If the pixel (u,v) is the floor surface, $\nu_{u,v}$ is approximately equal to $\left(\rho^{\prime },\varphi^{\prime },\theta^{\prime }\right)$. We therefore calculate Φ as follows:

(14) $$\Phi =\left\{\left[\begin{array}{c} u\\ v \end{array}\right]|\begin{array}{c} \rho^{\prime }-\eta_{\rho }<\nu_{u,v,\rho }<\rho^{\prime }+\eta_{\rho }\\ \cap \varphi^{\prime }-\eta_{\varphi }<\nu_{u,v,\varphi }<\varphi^{\prime }+\eta_{\varphi }\\ \cap \theta^{\prime }-\eta_{\theta }<\nu_{u,v,\theta }<\theta^{\prime }+\eta_{\theta } \end{array}\right\}.$$

Also, if the pixel (u,v) is the bed region, $\nu_{u,v}$ is approximately equal to $\left(\rho^{\prime \prime },\varphi^{\prime },\theta^{\prime }\right)$ of Section 2.3.3. We therefore calculate Ω as follows:

(15) $$\Omega =\left\{\left[\begin{array}{c} u\\ v \end{array}\right]|\begin{array}{c} \rho^{\prime \prime }-\eta_{\rho }<\nu_{u,v,\rho }<\rho^{\prime \prime }+\eta_{\rho }\\ \cap \varphi^{\prime }-\eta_{\varphi }<\nu_{u,v,\varphi }<\varphi^{\prime }+\eta_{\varphi }\\ \cap \theta^{\prime }-\eta_{\theta }<\nu_{u,v,\theta }<\theta^{\prime }+\eta_{\theta } \end{array}\right\}.$$

Red and blue in Figure 3b show the calculation results of Ω and Φ of the image (Figure 3a) using Equation (15) and Step 2, respectively. We used the following constants: δ=15 cm, $\eta_{\rho }=10\;\mathrm{cm}$, $\eta_{\varphi }=20{}^{\circ}$, $\eta_{\theta }=20{}^{\circ}$, $S_{\rho }=2\;\mathrm{cm}$, $S_{\varphi }=1{}^{\circ}$, and $S_{\theta }=1{}^{\circ}$.

##### Alternative 2: DDC Method at High Speed (DDC-S)

Recalculation for each pixel is not performed, and horizontal plane Φ is considered the floor surface if the Quadtree is used directly and following is satisfied:

(16) $$\Phi =\left\{\forall \left[\begin{array}{c} u\\ v \end{array}\right]\in Q_{i}|\begin{array}{c} \rho^{\prime }-\eta_{\rho }<\mu_{i,\rho }<\rho^{\prime }+\eta_{\rho }\\ \cap \varphi^{\prime }-\eta_{\varphi }<\mu_{i,\varphi }<\varphi^{\prime }+\eta_{\varphi }\\ \cap \theta^{\prime }-\eta_{\theta }<\mu_{i,\theta }<\theta^{\prime }+\eta_{\theta } \end{array}\right\}.$$

Similarly, Ω is considered a bed-region candidate if the Quadtree is used directly and following is satisfied:

(17) $$\Omega =\left\{\forall \left[\begin{array}{c} u\\ v \end{array}\right]\in Q_{i}|\begin{array}{c} \rho^{\prime \prime }-\eta_{\rho }<\mu_{i,\rho }<\rho^{\prime \prime }+\eta_{\rho }\\ \cap \varphi^{\prime }-\eta_{\varphi }<\mu_{i,\varphi }<\varphi^{\prime }+\eta_{\varphi }\\ \cap \theta^{\prime }-\eta_{\theta }<\mu_{i,\theta }<\theta^{\prime }+\eta_{\theta } \end{array}\right\}.$$

##### 2.3.5. Step 5: Calculation of the Bed-Region Ψ

Although red in Figure 3b indicates the bed-region candidate, there are some small red areas outside the bed because Ω is extracted not only for the bed surface but also for horizontal surfaces at the same height as the bed. We therefore implement the labeling method for Ω as in Section 2.2 and use a label with the largest area as the final bed region Ψ. Figure 3c shows the result of extracting Ψ from Figure 3b.

##### 2.3.6. Step 6: Automatic Calculation of the Sensor Attitude ***R*** and Distances *l* and *m*

Although it is possible to calculate the sensor attitude ***R*** and distances *l* and *m* directly using (ρ,φ,θ), the Hough transform generates quantization errors [36]. Instead, we calculate ***R***, *l*, and *m* by the PDC method (see Section 2.2), using Equations (5) through (9).

#### 2.4. Reconfiguration of the Bed Region

**Figure 4:**
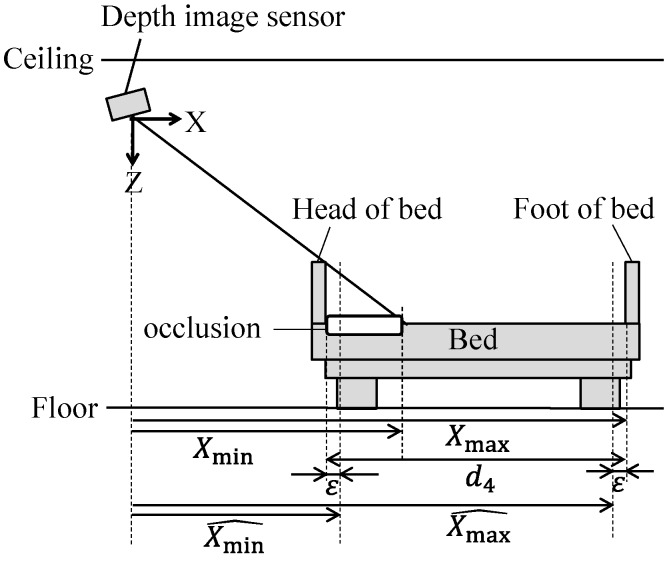
Occlusion by head and foot of bed.

**Figure 5:**
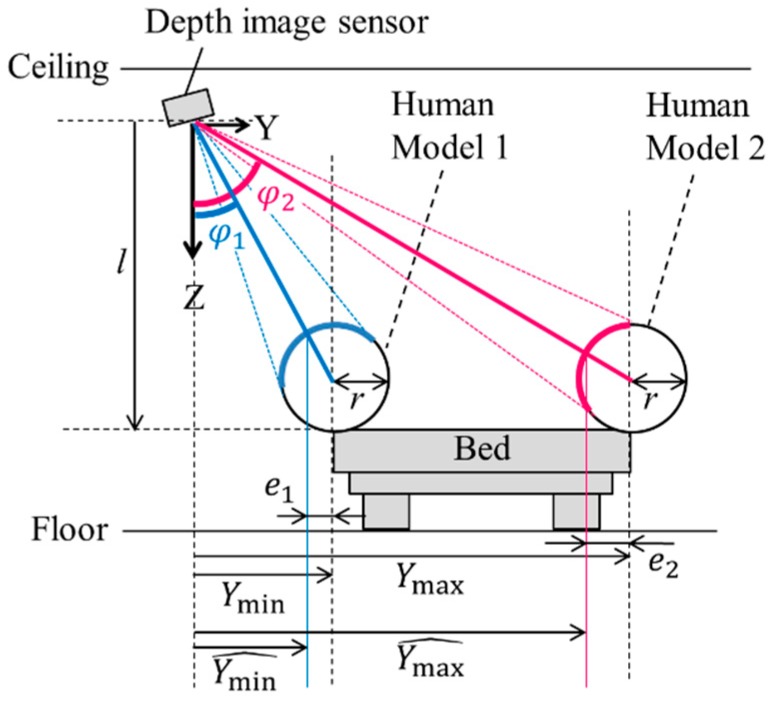
Difference between body surface and gravity center.

Figure 4 and Figure 5 show schematic views of the XZ and YZ axes cross sections (Figure 1a), respectively. The bed regions as shown in Figure 2e and Figure 3c seemed capable of being extracted, but part of the bed region is hidden by the head of the bed; this “occlusion,” shown in Figure 4, does not occur at the foot of the bed (in this case), as it happens where the obstruction is closer to the sensor end of the bed. Therefore, we measure the bed length *d*_4_ beforehand, and if the automatically calculated result for bed length is shorter than *d*_4_, the bed region is extended toward the sensor end. Also, in order to exclude the head and foot of the bed from the bed region, we shorten the bed region by 2*ε*, as illustrated in Figure 4.

The values of $\tilde{X}$ and $\tilde{Y}$ obtained by Equation (7) are used in Equation (18) to calculate bed edge coordinates *X*_min_, *X*_max_, *Y*_min_, and *Y*_max_, and the bed-edge coordinates (*X*_min_, *X*_max_, *Y*_min_, and *Y*_max_), and the bed region is expanded and contracted using Equations (19) and (20).

(18) $$\left[\begin{array}{c} X_{\min}\\ X_{\max}\\ Y_{\min}\\ Y_{\max} \end{array}\right]=\left[\begin{array}{c} \min\left(\tilde{X}|L_{X\min}<\tilde{X}<L_{X\max}\right)\\ \max\left(\tilde{X}|L_{X\min}<\tilde{X}<L_{X\max}\right)\\ \min\left(\tilde{Y}|L_{Y\min}<\tilde{Y}<L_{Y\max}\right)\\ \max\left(\tilde{Y}|L_{Y\min}<\tilde{Y}<L_{Y\max}\right) \end{array}\right],$$

(19) $$\hat{X_{\min}}=\{\begin{array}{c} X_{\max}-d_{4}+\varepsilon \;\;|X_{\min}>0\cap X_{\max}-X_{\min}<d_{4}\\ X_{\min}+\varepsilon \;\;\;\;\;\;\;\;\;\;\;\;|X_{\min}\leq 0\cup X_{\max}-X_{\min}\geq d_{4} \end{array},$$

(20) $$\hat{X_{\max}}=\{\begin{array}{c} X_{\min}+d_{4}-\varepsilon \;|X_{\max}<0\cap X_{\max}-X_{\min}<d_{4}\\ X_{\max}-\varepsilon \;\;\;\;\;\;\;\;\;\;|X_{\max}\geq 0\cup X_{\max}-X_{\min}\geq d_{4} \end{array},$$

where, $\hat{X_{\min}}$ and $\hat{X_{\max}}$ are bed-edge coordinates after expansion and contraction (Figure 4); and $L_{X\min}$, $L_{X\max}$, $L_{Y\min}$, and $L_{Y\max}$ are threshold values for excluding outliers. The latter (*L* values) are calculated using the interquartile method [37].

(21) $$\left[\begin{array}{c} L_{X\min}\\ L_{X\max}\\ L_{Y\min}\\ L_{Y\max} \end{array}\right]=\left[\begin{array}{c} f_{Q1}\left(\tilde{X}\right)-1.5\left(f_{Q3}\left(\tilde{X}\right)-f_{Q1}\left(\tilde{X}\right)\right)\\ f_{Q3}\left(\tilde{X}\right)+1.5\left(f_{Q3}\left(\tilde{X}\right)-f_{Q1}\left(\tilde{X}\right)\right)\\ f_{Q1}\left(\tilde{Y}\right)-1.5\left(f_{Q3}\left(\tilde{Y}\right)-f_{Q1}\left(\tilde{Y}\right)\right)\\ f_{Q3}\left(\tilde{Y}\right)+1.5\left(f_{Q3}\left(\tilde{Y}\right)-f_{Q1}\left(\tilde{Y}\right)\right) \end{array}\right],$$

where, *f_q_*_1_ and *f_q_*_3_ are functions to calculate the first and third quartiles, respectively.

Furthermore, since the depth sensor acquires the surface shape, the patient position acquired by the sensor may be slightly different from the actual gravity center of the patient. For example, if the patient protrudes halfway from the end of the bed on the sensor side (e.g., “Human Model 1” in Figure 5), the patient’s image is in the blue-arc position, mostly outside the bed. Conversely, if the patient protrudes halfway from the end of the bed opposite the sensor side (e. g., “Human Model 2” in Figure 5), the patient’s image is in the red-arc position, mostly inside the bed. As a result, if the patient gets out of or falls off the bed on the opposite side of the sensor, the alarm sensitivity will be poor.

To address this problem, we consider human models with a radius *r* that protrude halfway from the end of the bed as shown in Figure 5, where *r* is set in advance. Next, we calculate differences *e*_1_ and *e*_2_ between surface center positions and gravity-center positions of the human models using

(22) $$e_{1}=r\sin\varphi_{1}=r\sin\left(\sin^{-1}\frac{Y_{\min}}{\sqrt{{\left(l-r\right)}^{2}+Y_{\min}{}^{2}}}\right)=\frac{rY_{\min}}{\sqrt{{\left(l-r\right)}^{2}+Y_{\min}{}^{2}}},$$

and

(23) $$e_{2}=r\sin\varphi_{2}=r\sin\left(\sin^{-1}\frac{Y_{\max}}{\sqrt{{\left(l-r\right)}^{2}+Y_{\max}{}^{2}}}\right)=\frac{rY_{\max}}{\sqrt{{\left(l-r\right)}^{2}+Y_{\max}{}^{2}}}.$$

Finally, bed positions $Y_{\min}$ and $Y_{\max}$ are moved to $\hat{Y_{\min}}$ and $\hat{Y_{\max}}$ respectively using

(24) $$\hat{Y_{\min}}=Y_{\min}-e_{1},$$

and

(25) $$\hat{Y_{\max}}=Y_{\max}-e_{2},$$

where $\hat{X_{\min}}$, $\hat{X_{\max}}$, $\hat{Y_{\min}}$, and $\hat{Y_{\max}}$ are initial calibration variables; and $e_{1}$ and $e_{2}$ are the surface- and gravity-center differences, as calculated in Equations (22) and (23), respectively.

#### 2.5. Recognition of the Spatial Domain

In the actual monitoring process, using the previously calculated initial-calibration variables, we derive the world coordinate values $P_{u,v}=\left(X_{u,v},Y_{u,v},Z_{u,v}\right)$ by applying Equations (1) and (6) to each pixel (u,v) on the monitoring image. Then the eight spatial domains $D_{u,v}$ (Figure 1c) are calculated by

(26) $$D_{u,v}=\left\{\begin{array}{cc} \text{I} & |E_{u,v}=1\cap Z_{u,v}\leq l-d_{2}\\ \text{II} & |E_{u,v}=1\cap l-d_{2}<Z_{u,v}\leq l-d_{1}\\ \text{III} & |E_{u,v}=1\cap l-d_{1}<Z_{u,v}\\ \text{IV} & |E_{u,v}=0\cap Z_{u,v}\leq l-d_{2}\\ \text{V} & |E_{u,v}=0\cap l-d_{2}<Z_{u,v}\leq l-d_{1}\\ \text{VI} & |E_{u,v}=0\cap l-d_{1}<Z_{u,v}\leq l\\ \text{VII} & |E_{u,v}=0\cap l<Z_{u,v}\leq m-d_{3}\\ \text{VIII} & |E_{u,v}=0\cap m-d_{3}<Z_{u,v} \end{array}\right\},$$

where $E_{u,v}$ is a variable indicating whether location is inside or outside the bed region (1 or 0, respectively) as

(27) $$E_{u,v}=\left\{\begin{array}{ll} 1 & |\hat{X_{\min}}\leq X_{u,v}\leq \hat{X_{\max}}\cap \hat{Y_{\min}}\leq Y_{u,v}\leq \hat{Y_{\max}}\\ 0 & |X_{u,v}\langle \hat{X_{\min}}\cup X_{u,v}\rangle \hat{X_{\max}}\cup Y_{u,v}\langle \hat{Y_{\min}}\cup Y_{u,v}\rangle \hat{Y_{\max}} \end{array}\right\}.$$

Finally, the number of pixels (area) included in each domain is counted, and automatic detection of action such as sitting up in bed or exiting the bed is performed by using the area variation of each domain.

## 3. Experimental Results and Discussions

### 3.1. Experimental Methods

**Figure 6:**
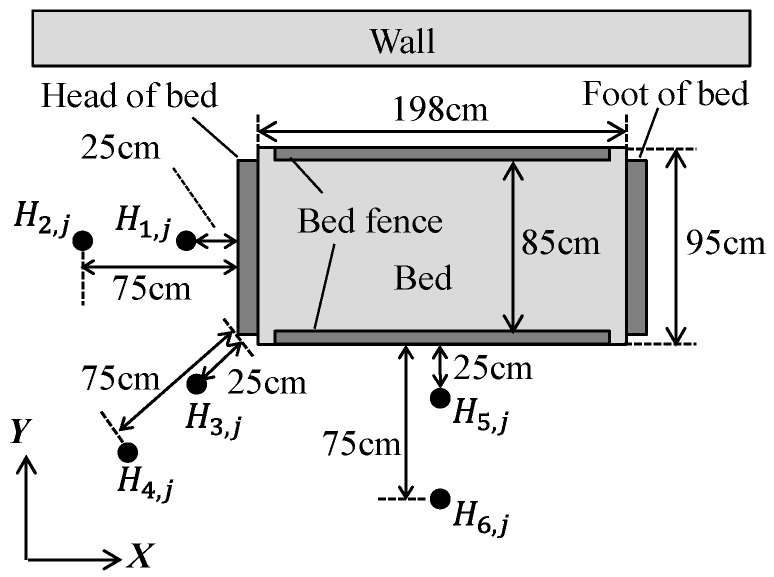
Arrangement of sensors.

We devised experimental methods to verify the effectiveness of our proposed method for bed monitoring. First, we set the ASUS XTION PRO depth sensor (resolution: 320 × 240 pixel) at 18 viewpoints H_1,*j*_ to H_6,*j*_ (six *X*–*Y* locations, at three heights from the floor: *j* = 1.5, *j* = 1.8, and *j* = 2.0 m) (Figure 6) and take depth images with the bed fence down. (The height difference between the bed surface and the bed fence is 4 cm) Next, we conducted the pre-experiments for examining the optimal parameters of the Canny algorithm of the PDC method in Section 2.2. The results are described in Section 3.2. Afterwards, we compare the calibration precision and time of the three methods (PDC, DDC-D, and DDC-S) described earlier in Section 2, by running Experiments 1 through 4 below. We also verified the previously discussed reconfiguration of the bed region and recognition of the spatial domain in Experiments 5 and 6 below.

Since the PDC method recognizes a label having the maximum area as a bed area, the bed area must be taken larger than the floor area. Also, our system requires is that both the bed and floor are taken and this total area is larger than the wall area. Therefore, in the above Experiments 1 through 6, the sensor was installed so as to satisfy these conditions. On the other hand, in order to verify these conditions themselves, we experimented without satisfying these conditions in Experiment 7. These methods are shown in Section 3.1.7 and results are shown in Section 3.5.

The experiments in this section are accuracy verification experiments of the proposed method. Although we should compare with some conventional methods, conventional methods listed in this paper [23,24,26] did not have detailed designs sufficient to reproduce; therefore, we excluded the comparison with the conventional method.

#### 3.1.1. Experiment 1

The height of the bed as measured (46 cm) is compared to the value calculated, which is the average of 18 viewpoints of (*m* − *l*). The standard deviation of (*m* − *l*) is also calculated and discussed.

#### 3.1.2. Experiment 2

The width of the bed as measured (95 cm) is compared to the value calculated, which is the average of the bed width $\left|Y_{\max}-Y_{\min}\right|$ obtained during calibration. The standard deviation of $\left|Y_{\max}-Y_{\min}\right|$ is also calculated and discussed.

#### 3.1.3. Experiment 3

The image coordinates set ***K*** of the four corners of the bed is calculated using

(28) $$B=\left[\begin{array}{cccc} X_{\min} & X_{\min} & X_{\max} & X_{\max}\\ Y_{\min} & Y_{\max} & Y_{\min} & Y_{\max}\\ l & l & l & l \end{array}\right],$$

and

(29) $$B^{\prime }=R^{-1}B,$$

where, ***B*** and ***B’*** are the coordinates set of the four bed corners in the world coordinate and sensor coordinate systems, respectively, and

(30) $$K=\left[\begin{array}{ccc} 1 & 0 & 0\\ 0 & -1 & 0 \end{array}\right]\frac{1}{a\tilde{Z}B^{\prime }}B^{\prime }+\left[\begin{array}{cccc} C_{u} & C_{u} & C_{u} & C_{u}\\ C_{v} & C_{v} & C_{v} & C_{v} \end{array}\right].$$

Next, we visually evaluate whether ***K*** properly indicates the bed corners, according to the following criteria:

- 3 points when ***K*** correctly indicates the actual corner points using default parameters.
- 2 points when ***K*** correctly indicates the actual corner points after changing the parameters.
- 1 point when ***K*** deviates from the actual corner points even if any parameter is used.
- 0 point when ***K*** does not indicate the actual corner points.

The default parameters are results of empirical calculations for the highest precision and are the same as in the “Calibration Method” section. Specifically, we used the range of neighboring pixels *b* = 5 in Equation (2), the number of clusters *k* = 4 in the k-means++ algorithm, the Quadtree’s recursive-division criterion *s_t_* = 2 cm, the lower limit of the height of the bed *δ* = 15 cm in Equation (12), the extraction thresholds of the horizontal plane $\eta_{\rho }=10\;\mathrm{cm}$, $\eta_{\varphi }=20{}^{\circ}$, and $\eta_{\theta }=20{}^{\circ}$, and voxel sizes $S_{\rho }=2\;\mathrm{cm}$, $S_{\varphi }=1{}^{\circ}$, and $S_{\theta }=1{}^{\circ}$. The Canny parameters $T_{1}$, $T_{2}$, and σ will be examined in the pre-experiment in Section 3.2.

#### 3.1.4. Experiment 4

Programs are built using only single-thread CPU in the following environment, and then the processing times of the three methods (PDC, DDC-D, DDC-S) are compared:

- PC: Desktop computer, MDV-GX9200B made by Mouse Computer Co., LTD. equipped with Windows 8.1.
- CPU: Intel Core i7-4820K 3.70 GHz
- RAM: 32.0 GB
- Development environment: Microsoft Visual C++ 2010
- Library: OpenCV 2.4.2 and OpenNI

#### 3.1.5. Experiment 5

***K*** is recalculated by replacing *X*_min_, *X*_max_, *Y*_min_, and *Y*_max_ in Equations (28)–(30) with $\hat{X_{\min}}$, $\hat{X_{\max}}$, $\hat{Y_{\min}}$, and $\hat{Y_{\max}}$ respectively; the parameters *r* and *ε* are set to 20 and 10 cm, respectively; and the bed length *d*_4_ is set to 198 cm from actual measurement. Whether the method described in the section “Reconfiguration of the Bed Region” yields the expected result is verified visually.

#### 3.1.6. Experiment 6

The motion of a patient’s getting out of bed is simulated, and we take the depth images of the motion at 5 frames per second using the sensor installed at the viewpoint *H*_4,2_ (Figure 6). Thresholds *d*_1_, *d*_2_, and *d*_3_ are set to 15, 50, and 30 cm, respectively, and then the spatial domain $D_{u,v}$ is calculated for all images. Whether the method described in the section “Recognition of the Spatial Domain” is correct is verified visually.

In this paper, given that we focus on initial calibration, verifications of *r*, *d*_1_, *d*_2_, and *d*_3_ are excluded.

#### 3.1.7. Experiment 7

In order to confirm the limitations of our method, we experiment whether initial calibrations are possible under the following two specific conditions.

- Install and take an image so that the floor area is larger than the bed area.
- Install and take an image so that the wall area is larger than the sum of the bed and floor areas.

In both cases, we installed the depth sensor at the position $H_{4,1.5}$ in Figure 6 and took depth images with the sensor tilted.

### 3.2. Results of Pre-Experiment

In order to investigate the optimal parameters of the Canny algorithm of the PDC method in Section 2.2, we tried edge extractions under the following conditions for depth images (value range 0–255) taken from 18 viewpoints in Figure 6.

- $T_{1}=\left\{0,\;50,\;100\right\}$.
- $T_{2}=\left\{50,\;100,\;150,\;200\right\}$, where $T_{2}>T_{1}$.
- σ={3, 5}.

**Figure 7:**
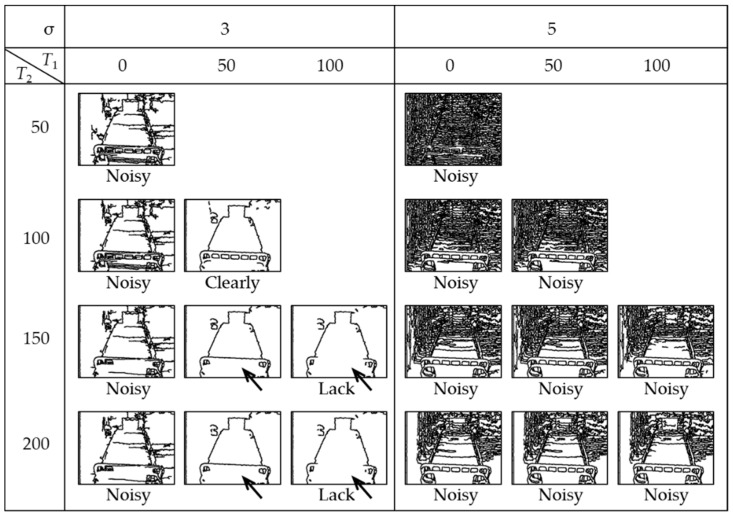
Pre-experimental results for analyzing optimal parameters of the Canny algorithm.

Figure 7 shows processing results of captured images at the viewpoint of $H_{2,1.5}$. In all cases where σ=5, too many lines were extracted (noisy). When σ=3 and $T_{1}=0$, the results were slightly noisy. When σ=3 and $T_{1}=100$, the bed heads were lacking (arrow parts). When σ=3, $T_{1}=50$, and $T_{2}=\left\{150,\;200\right\}$, although they did not affect the calibration, the bed heads were slightly lacking (arrow parts). Therefore, when σ=3, $T_{1}=50$, and $T_{2}=100$, the edge could be extracted most clearly. Since the same results were obtained from the other 17 viewpoints, we used the above values in Experiments 1 to 7. Note that the optimum parameters for the Canny algorithm may vary slightly depending on the type of depth sensor used.

### 3.3. Results and Discussion of Calibration Experiments (Experiments 1–4)

**Figure 8:**
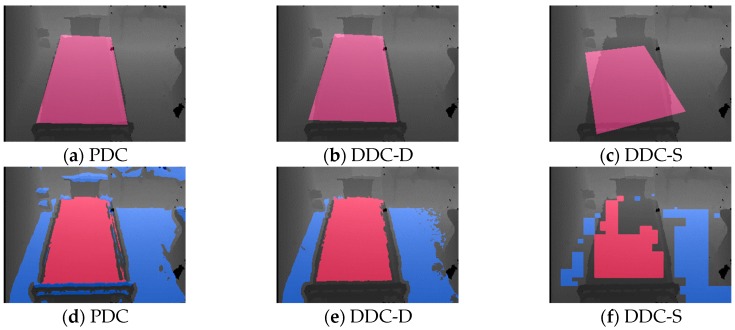
Process of calibration by three methods. (**a**) through (**c**) show bed regions as rectangles made by ***K***. (**d**) shows bed-region candidate Ω and floor surface *Φ*, and both (**e**) and (**f**) shows bed region *Ψ* and floor surface *Φ*.

The results of the calibration experiments (1 through 4) are shown in Table 1 and Figure 8. Across all three calibration methods (PDC, DDC-D, and DDC-S), the average values for Experiments 1 and 2 were close to the measured values 46 and 95 cm, respectively, and there were no significant differences among the three methods. Also, the standard deviations for Results 1 and 2 were about 1 and 5 cm, respectively, and there were no significant differences among the three methods. Since the resolution of the sensor used in the experiment is about 2 cm, all three methods were able to acquire the bed height and width with accuracy close to sensor resolution.

In Result 3, only DDC-S yielded a low point score. To investigate this, we examined the data progression during the calibration process. Figure 8 shows an example of the process of calibration of the image acquired at viewpoint *H*_2,2_, and Figure 8a–c show the results of the three methods of drawing the bed region as rectangles made by calculating ***K***, using Equation (30). Figure 8d shows the result of drawing Ω and *Φ* regions by the PDC method, and Figure 8e,f respectively show the drawing results of *Ψ* and *Φ* regions by the DDC-D and DDC-S methods. The red color in each image represents the bed surface *Φ*. However, note that the blue color in Figure 8e,f represents only the floor surface *Ψ*, whereas that in Figure 8d represents all the Ω horizontal planes, including the floor, shelf, and other surfaces. Figure 1b, Figure 2 and Figure 3 also show progressive data for images acquired at viewpoint *H*_2,2_. In Figure 8d,e, the bed regions were extracted appropriately at the pixel level. However, in Figure 8f, because DDC-S is an algorithm to extract the regions at the block level, the bed region was not extracted at the pixel level. We assume that this adversely affected the bed position calculation, as shown in Figure 8c, and lowered the visual-observation score for Result 3 in Table 1.

For the three methods, calibration time (Result 4 in Table 1) was the fastest for DDC-S and slowest for PDC. Initial calibration is performed only once after installing the sensor, and robustness is more important than calculation speed in many cases. Therefore, DDC-D and PDC are usually more effective than DDC-S.

In this paper, we experimented with a depth sensor with a resolution of 320 × 240 pixels and obtained calibration accuracy of several centimeters. In bed monitoring, it will be possible to operate with this accuracy. On the other hand, depth sensors with higher resolution have been developed in recent years and we can be expected to improve accuracy by using them although processing time will increase.

### 3.4. Results and Discussion of Bed-Region and Spatial-Domain Experiments (Experiments 5–6)

**Figure 9:**
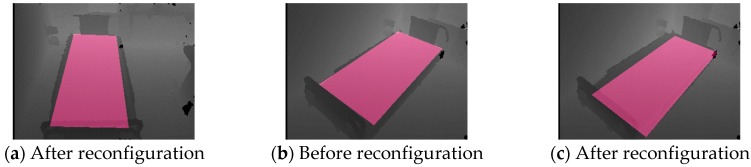
Bed regions calculated by DDC-D method. (**a**) was acquired at viewpoint *H*_2,2_, and (**b**) and (**c**) at viewpoint *H*_4,2_.

**Figure 10:**
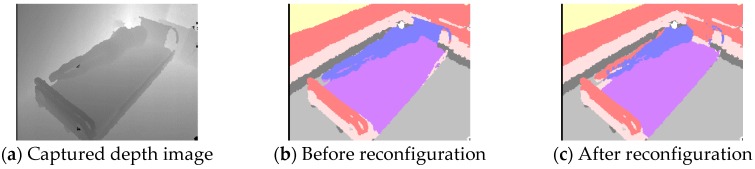
Experimental result of motion simulation.

The results of bed-region-reconfiguration and spatial-domain-recognition experiments (5 and 6) are shown in Figure 9 and Figure 10. The result of reconfiguration of the bed region imaged in Figure 8b and calculated using the method described in the section “Reconfiguration of the Bed Region” is shown in Figure 9a. In this reconfiguration, the bed region spread to include more of the lower sides than in Figure 8b and included the region at the foot of the bed. Figure 9b,c show the results of calculation of the bed region before and after reconfiguration for images acquired at another viewpoint, *H*_4,2_. As expected, the bed region shown in Figure 9c is extended more to the lower right (toward the sensor) than the one of Figure 9b. All the other viewpoints also produced the expected results. These outcomes suggest that the calculations in Equations (18) through (21) are effective for solving the problem of occlusion by the head of the bed.

Subsequently, we simulated a state of being in the lateral decubitus position on the edge of the bed, acquired the depth image at the viewpoint *H*_4,2_, and carried out the verification experiment by applying the spatial-domain calculation Equation (26). Figure 10a shows the captured depth image, and Figure 10b,c respectively show segmentation images before and after reconfiguration of the bed by applying calculations in Equations (19), (20), (24), and (25). For each pixel in Figure 10b,c, regions I to VIII were colored with light blue, blue, purple, yellow, red, light pink, dark gray, and light gray, respectively. Note that black indicates defective pixels and white indicates saturated pixels which are located farther than the photographable region and the exact position cannot be calculate.

In Figure 10b, most of the body was determined to be in the bed (blue) despite simulating the state in which part of the body protruded outside the bed. In contrast, in Figure 10c, about half of the body was determined as outside the bed (red). These results suggest that the calculations in Equations (22) through (25) are effective for reconfiguration of the spatial domain considering the gravity center of the patient. In addition, whereas the head of the bed in Figure 10b is mostly region II (blue), it is shown as mostly region V (red) in Figure 10c. The effect of *ε* (see Figure 4), this makes it possible to limit region II to only the identification of the presence or absence of a patient.

### 3.5. Results and Discussion of Two Specific Condition Experiments (Experiment 7)

**Figure 11:**
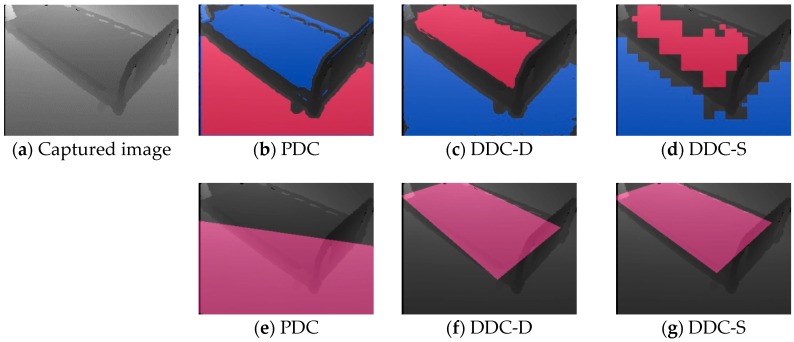
Experimental results (**a**) when the floor area is larger than the bed area. The red and blue colors in (**b**) to (**d**) represent regions extracted as the bed surface Φ and the floor surface Ψ, respectively. The red colors in (**e**) to (**g**) represent the bed regions before correction calculated in the same way as in Section 3.4.

This section describes the calibration results of depth images taken under the two specific conditions. Figure 11a shows a depth image taken by setting up so that the floor area is larger than the bed area. Figure 11b–d respectively shows the initial calibration results of Figure 11a using the PDC, DDC-D, and DDC-S, and their red and blue colors respectively represent regions extracted as the bed surface Φ and the floor surface Ψ. Figure 11c,d succeeded in extraction because they were colored as expected, however Figure 11b failed because red and blue were opposite. Figure 11e–g respectively represents bed regions before the correction calculated based on Figure 11b–d. These results also showed that DDC-D and DDC-S succeeded but PDC failed. The calculated bed heights of DDC-D and DDC-S were 44.9 and 45.8 cm, respectively, which were close to the true value (46 cm). Similarly, the calculated bed widths of them were 99.3 and 98.3 cm, respectively, which were also close to the true value (95 cm). These results show that the bed area must be larger than the floor area in the PDC as described in Section 2.2, however DDC-D and DDC-S can be calibrated under these conditions.

**Figure 12:**
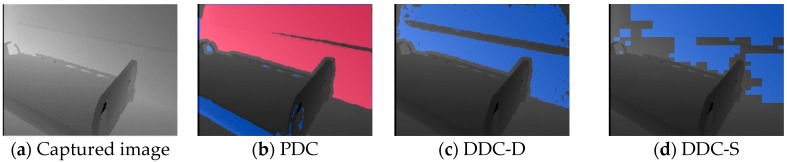
Experimental results (**a**) when the wall area is larger than the sum of the bed and floor areas. The red and blue colors in (**b**) to (**d**) represent regions extracted as the bed surface Φ and the floor surface Ψ, respectively.

Next, we installed and took an image so that the wall area was larger than the sum of the bed and floor areas. Figure 12a shows the captured depth image, and Figure 12b–d shows the results of calibration with the PDC, DDC-D, and DDC-S, respectively. Similar to Figure 11, their red and blue colors respectively represent regions extracted as the bed surface Φ and the floor surface Ψ. In Figure 12b, the back wall was recognized as a bed region. Also, in Figure 12c,d, the back walls were recognized as floor regions. Therefore, as expected, both our methods do not work under this condition.

## 4. Conclusions

We proposed a calibration method for a bed-monitoring system using an infrared-image depth sensor. This method can calculate the bed region, floor region, and sensor position and attitude automatically and robustly. The system using our method can recognize how elevated a patient is and whether the patient is in or out of the bed, even when the sensor is attached in any position and attitude without installation on the ceiling, such as on a tripod or on a bed fence.

For the recognition of the bed and floor regions, we propose three methods—the PCA-based depth-sensor calibration (PDC) method and the two D-KHT-based depth-sensor calibration methods characterized by either high density (DDC-D) or high speed (DDC-S). The PDC method is based on PCA, k-means++ clustering, and the Canny edge-detection method. Both the DDC-D and DDC-S methods are based on the D-KHT plane-detection method. The DDC-S method estimates the bed and floor regions directly using the plane-detection results, whereas the DDC-D method repeatedly calculates both regions for each pixel after applying D-KHT. Experimental results show that DDC-S calibrates at high speed but with low robustness, and although PDC and DDC-D require more calibration time than DDC-S, their robustness is higher. In many cases, since initial calibration does not require real-time processing, PDC or DDC-D are more useful than DDC-S.

However, the PDC method must be taken so that the bed area is larger than the floor area. Also, our system requires that both the bed and floor are taken and these total area is larger than the wall area. We also experimented with these specific conditions and confirmed that the calibration failed. If the calibration fails during actual use, it is necessary to change the orientation of the sensor or change the installation position.

Furthermore, we proposed a method of reconfiguration of the spatial domain that considers both occlusion by the head (or foot) of the bed and the gravity center of the patient. Experimental results of multi-view calibration and motion simulation show that this method is effective for spatial-domain recognition.

Future work will include improved timing of detection of a patient’s rising or sitting up, getting out of bed, falling, and other potentially dangerous movements as well as verification of effectiveness in actual hospital and other care facilities.

## Figures and Tables

**Table 1:** Results of calibration Experiments 1 through 4.

		PDC	DDC-D	DDC-S
1. Height of bed (cm)	Average	46.09	45.94	45.96
	Standard deviation	0.93	0.92	1.05
2. Width of bed (cm)	Average	94.25	96.29	93.72
	Standard deviation	4.64	5.15	5.39
3. Visual observation (point)	Average	3.00	2.94	2.06
4. Calibration time (ms)	Average	1400	964	243
	Standard deviation	110	71	65
